# Influence of Water on Chemical Vapor Deposition of Ni and Co thin films from ethanol solutions of acetylacetonate precursors

**DOI:** 10.1038/srep18194

**Published:** 2015-12-14

**Authors:** Theodor Weiss, Volkmar Zielasek, Marcus Bäumer

**Affiliations:** 1 Institut für Angewandte und Physikalische Chemie, Universität Bremen, Leobener Straße UFT, D-28359 Bremen, Germany

## Abstract

In chemical vapor deposition experiments with pulsed spray evaporation (PSE-CVD) of liquid solutions of Ni and Co acetylacetonate in ethanol as precursors, the influence of water in the feedstock on the composition and growth kinetics of deposited Ni and Co metal films was systematically studied. Varying the water concentration in the precursor solutions, beneficial as well as detrimental effects of water on the metal film growth, strongly depending on the concentration of water and the β-diketonate in the precursor, were identified. For 2.5 mM Ni(acac)_2_ precursor solutions, addition of 0.5 vol% water improves growth of a metallic Ni film and reduces carbon contamination, while addition of 1.0 vol% water and more leads to significant oxidation of deposited Ni. By tuning the concentration of both, Ni(acac)_2_ and water in the precursor solution, the fraction of Ni metal and Ni oxide in the film or the film morphology can be adjusted. In the case of Co(acac)_2_, even smallest amounts of water promote complete oxidation of the deposited film. All deposited films were analyzed with respect to chemical composition quasi *in situ* by XPS, their morphology was evaluated after deposition by SEM.

Chemical Vapor Deposition (CVD) and its variants such as Atomic Layer Deposition (ALD) are key technologies in the industrial production of thin metal films for a broad range of applications^1^. In the CVD family of processes a solid film is deposited from a vapor of precursor molecules by chemical reactions occurring on or in the vicinity of a substrate surface, which is usually heated to thermally stimulate the reaction. Metal CVD can be used to produce fibers, monoliths, foams, and powders of pure metals or alloys with free variation of the composition for catalysis, microelectronic, and optical devices^2^,^3^. There are two classes of organic precursors which are routinely used in CVD: organometallic (OM) precursors with metal centers mostly bonded to carbon and metal-organic (MO) precursors such as the class of β-diketonate complexes including the acetylacetonates (acac), hexafluoroacetylacetonates (hfac), dipivaloylmethanates (dpm), and alkoxides of different metals, i.e., with metal centers bonded to typically oxygen, nitrogen or sulfur^3^,^4^. Elucidating the CVD reaction mechanisms for these precursors comprehensively and in detail is prerequisite for process optimization and beneficial for the selection and synthesis of new precursors. In this respect, also boundary conditions may play important roles but often lack systematic studies.

One of the big problems using solid organic precursors in metal CVD is their sensitivity to air and moisture^5^,^6^,^7^. Generally, the hygroscopic nature of many OM and MO precursors promotes the formation of hydrates which leads to significant configuration changes within the precursor depending on the water content. For example, anhydrous Ni(acac)_2_ forms trimers in order to achieve an octahedral coordination around each nickel atom. When water is present, each Ni(acac)_2_ prefers to take up position in an octahedral complex with two water molecules for that purpose^7^,^8^,^9^,^10^. Such changes may induce strong dependencies of the precursor volatility and thermal stability on the water concentration^7^,^10^,^11^. Additionally, water may influence the precursor adsorption selectivity by formation of OH groups on the substrate surface and affect reaction mechanisms thereon^3^,^9^,^12^,^13^,^14^. Overall, the sensitivity of the precursor to moisture may render the control of parameters such as nucleation rate, growth rate, or precursor fragmentation difficult and thus affect quality (morphology) and purity (carbon incorporation) of the CV deposits.

Several studies have reported that water affects the reduction mechanisms of β-diketonates, in particular metal (M) (acac)_x_ precursors, employed in classical CVD and ALD regimes. M(acac)_x_ as CVD precursors have been in the focus of interest because they are commercially available low-cost products of minor toxicity and exhibit low evaporation temperatures and easily controllable purity^7^,^15^. Only few systematic studies on the influence of water on β-diketonates under CVD conditions are available and their results often appear to be contradictory. For instance, it has been reported that CVD with a β-diketonate such as Cu(hfac)_2_ as precursor in combination with H_2_ as reducing agent can be area-selective, i.e., copper, in this case, is deposited on metal substrates but not on oxide areas^16^,^17^,^18^,^19^. When adding water to the reaction gas mixture, some authors found a loss of the precursor’s selectivity to metal substrates^19^ while others reported, for apparently similar experiments, that the selectivity is unaffected^20^. Furthermore, there are several reports on an *increase* of carbon contamination due to the presence of water in the gas phase during metaloxide (MOx) deposition on soda lime glass substrates from Cu and Ni β-diketonates such as M(acac)_2_, M(dmg)_2_, and M(hfac)_2_^3^,^7^,^9^,^21^. Using the same β-diketonate precursors for obtaining M, MOx, and metalnitride films on SiO_2_, α-Al_2_O_3_ and polyimide substrates, others found, however, a *decrease* of carbon content when water was added to the CVD reactants mixture^22^,^23^,^24^. Finally, Utraininen *et al*. found as a general trend that H_2_O in combination with β-diketonate complexes like Cu/Ni/Pt (acac)_x_ (deposited on glass, SiOx, AlOx, and TiOx) leads to the deposition of metal oxide films during ALD^9^,^11^. In accordance with this trend, Borgharkar *et al*. showed for CVD from Cu(hfac)_2_ on TiN-coated Si(100) substrates that the metal film growth rate and the electrical conductivity of the deposit can be improved by *eliminating* H_2_O from the starting precursor^25^. In contrast, several other CVD studies (on many different types of substrates including Kapton) reported that *addition* of water enhanced the metal film nucleation and increased the rate of β-diketonate reduction by H_2_^16^,^17^,^22^,26–28.

In practice, controlling water contamination in the entire CVD process is harder than just to care for pure precursor feedstock. As pointed out by Pierson^2^, a pure reactant can become contaminated in the distribution system to the reactor by, amongst others, moisture even if gas-tight metal lines are used. Therefore, in order to limit costs it is essential to know what grades of purity of precursors, feed gases and reactor lines have to be maintained for good results. Also, as demonstrated above, water may even have beneficial effects on the composition or growth rate of the deposit. Consequently, in order to make use of these effects purposefully and reach optimal growth conditions with reasonable efforts, systematic studies on the influence of water on CVD processes are, as also noted by others^21^, a prerequisite.

In the following we will present a comprehensive study of the influence of water on metal film formation exemplarily in a special field of CVD: the pulsed-spray evaporation (PSE)-CVD from a liquid feedstock of acetylacetonate precursors in ethanol which serves as solvent and reducing agent at the same time^29^,^30^,^31^,^32^. The PSE-CVD method offers several advantages compared to the classical gas phase CVD: Metal films can be obtained without employing gaseous hydrogen, there is less demand on the precursor in terms of volatility and stability, and the precursor does not need to be heated for spray evaporation^29^,^32^. The selection of acetylacetonates as precursor was not only based on low costs, low toxicity, and the commercial availability of a wide variety of different metals (as mentioned above) but acetylacetonates are also readily soluble in ethanol. To a liquid, alcohol-based precursor solution, water is easily added and its amount can be precisely varied, allowing for rigorous studies of the influence of water on the CVD process. By varying systematically and in a broad range the water content in Ni and Co acetylacetonate precursors for PSE-CVD of Ni and Co, we found both, beneficial and detrimental effects of water on the metal film growth, strongly depending on the concentration of water and the β-diketonate in the precursor solution. All deposited films were analyzed with respect to chemical composition quasi *in situ* by x-ray photoelectron spectroscopy (XPS) and their morphology was evaluated after deposition by scanning electron microscopy (SEM).

## Experimental Methods

The deposition experiments were performed in a home-built PSE-CVD reactor described in detail elsewhere^32^. In brief, the reactor is directly attached to an ultra-high vacuum system with XPS (Multiprobe system, Omicron Nanotechnology, base pressure 10^−10^ mbar) and allows for contamination-free transfer of a sample from the reactor to the XPS after any stage of deposition. Apart from the water concentration, all experimental deposition parameters (see Supplementary Table S1 available online) were chosen on the basis of previous publications^29^,^30^,^33^,^34^. For all depositions discussed in the following, solutions of commercially available anhydrous 95% Ni(acac)_2_ and 97% Co(acac)_2_ (Sigma-Aldrich) in absolute ethanol (VWR Chemicals) were used. In PSE-CVD the concentration of the β-diketonate precursor molecule in the liquid feedstock is an adjustable parameter. To obtain a feedstock with 2.5 mM Ni concentration, 64.2 mg of the Ni precursor was weighed and dissolved in 100 ml alcohol. The solution was ultrasonically shaken to achieve complete dissolution. With double and triple amounts of Ni precursor, also 5.0, and 7.5 mM Ni precursor feedstock were prepared. Using 192.9 mg of Co(acac)_2_, a 7.5 mM Co precursor feedstock was produced in a similar way. All precursor solutions were prepared freshly directly before PSE-CVD. The purity of the absolute ethanol (>99.9 vol%) was confirmed by an analytical hydrometer. Further tests showed that keeping the absolute ethanol in contact with the atmosphere for one week resulted in absorption of water up to 3 vol%. Therefore, fresh preparation of the precursor solutions directly before PSE-CVD was essential to ensure the purity of our experiments and neutralize the “aging” effect of the precursor feedstock. For a systematic study of the water influence on the CVD process, distilled H_2_O was added to the precursor solution to adjust the water concentration. It was varied from 0.0 vol% to 15.0 vol% (1.0 vol% corresponds to 0.555 mol/l) and could be controlled within ± 0.1 vol% as confirmed by the analytical hydrometer.

A piece from a Si(100) wafer was cut as substrate, cleaned with isopropanol in an ultrasonic bath, and dried in air, so that Ni or Co were deposited on top of the native or thermal silicon oxide layer of the wafer. XPS showed that the mild cleaning procedure left, very reproducibly, a level of 9 atom% residual carbon contamination on the substrate surface. In order to keep the substrate preparation simple, neither chemical cleaning steps nor in vacuo thermal treatments were employed to remove all carbon or the SiOx layer from the Si wafer. It should be noted that, depending on CVD applications, typically more vigorous substrate treatments are employed. For precursor delivery via the spray nozzle a solenoid valve pulse frequency of 2 Hz and a spray injection phase of 15 ms were used, meaning that the solenoid valve opened two times per second for 15 ms and a total deposition time of 30 min corresponded to a net precursor solution injection time into the reactor of 54 s. For all experiments shown in the following, the total deposition time was kept constant, resulting in different thicknesses of the deposits for different precursor concentrations, as will be detailed below. During deposition, the pressure in the deposition area as well as the sample surface temperature were adjusted and stabilized (see the previous publication for further details)^32^. The temperature for deposition was chosen equal to the optimal metal deposition temperature with the maximal growth rate determined in previous PSE-CVD experiments as 270 °C and 310 °C for Ni and Co precursors, respectively^29^,^32^,^34^. With one exception to be discussed below, higher temperatures were not employed to avoid undesirable decomposition of precursor ligands which would increase contamination of the deposited films. Below 260 °C, cobalt carbide formation was observed^34^, leaving a rather narrow temperature range for metal PSE-CVD for both, Ni and Co.

After the deposition process was finished, samples were transferred immediately *in vacuo* into UHV for XPS analysis. For XPS, non-monochromatized Al Kα radiation was used for photoelectron generation and a Leybold EA 10 Plus hemispherical energy analyzer with single-channeltron detector was employed. The obtained XP spectra were normalized and then fitted using Igor Pro (from Wave Metrics) and FITT (Seoul National University) software. For the fit a Shirley-type background was taken into account. The morphology of the deposited films was assessed *ex situ* using a Carl Zeiss Ultra Plus Field Emission scanning electron microscope operated at 15.0 keV electron energy.

## Results and Discussion

Conditions for growing (metallic) Ni films by PSE-CVD from Ni(acac)_2_ dissolved in ethanol had been established in previous work which reported Ni(acac)_2_ concentrations in the range from 2.5 to 10.0 mM as optimal and the growth rate as increasing linearly with the precursor concentration in that range^29^. Further increase of the Ni(acac)_2_ concentration is limited by the short lifetime (re-crystallization) of highly concentrated precursor solutions. Below 2.5 mM, film growth becomes impractically sluggish. For our own experiments we selected three different Ni(acac)_2_ concentrations (2.5, 5.0, and 7.5 mM) with sufficient precursor lifetime within the optimal range and performed PSE-CVD. As noted before, the total deposition time for all Ni films studied in the following was 30 min. The first part of this section will provide a full account of the observed effects of water concentration variation in the precursor solution on composition and morphology of the deposited Ni films. In the second part of this section, the influence of water on films deposited from 7.5 mM Co(acac)_2_ precursor solution will be demonstrated and discussed. In contrast to PSE-CVD of Cu or Ni, finding parameters for deposition of pure metal Co films from Co(acac)_2_ has been reported as difficult because cobalt precipitates mainly unreduced as carbide or oxide^35^. Moisture may play a key role here.

### Ni(acac)_2_ in EtOH on SiOx/Si(100)

**Figure 1:**
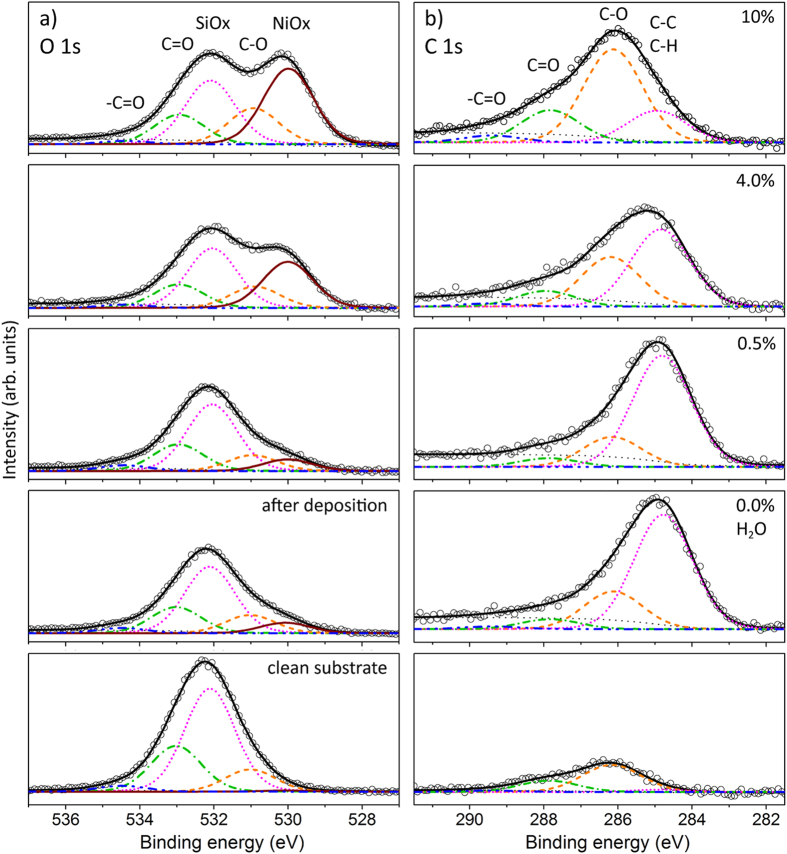
Fits of the O 1s (a) and C 1s (b) XPS spectra obtained from the SiOx/Si(100) substrate (bottom panels) and Ni/NiOx films grown ontop by PSE-CVD from 2.5 mM Ni(acac)_2_ in ethanol and with various water concentrations (0.0%, 0.5%, 4.0%, and 10.0 vol%) in the precursor solution at a substrate temperature of 270 °C.

The presentation of results and their discussion will be based on a quantitative analysis of XP spectra from the deposited films. Exemplarily, Fig. 1 depicts a selection of O 1s and C 1s XP spectra obtained in the take-off direction normal to the surface after PSE-CVD with 2.5 mM Ni(acac)_2_ precursor solution and shows the results of the fitting routine including all individual contributions that were considered for the fit. Examples of Ni 2p spectra and their fit by contributions from Ni^0^ and Ni^2+^ can be found elsewhere^32^. Based on a previous XRD study which found no evidence for Ni_x_C after PSE-CVD from Ni(acac)_2_ in ethanol for substrate temperatures in the range from 190 °C to 290 °C^36^, the presence of nickel carbide can be ruled out.

The solid (brown) curve labeled “NiOx” in Fig. 1a designates O 1s emissions with a binding energy of 529.9 eV stemming from Ni^2+^O^2–^ 37,38,39. It is absent on the clean substrate but can be detected after film deposition and increases with the water concentration in the liquid precursor feedstock. The dotted magenta “C-C/C-H” curve in Fig. 1b (highest intensity at 0.0 vol% water concentration) denotes C 1s emissions from carbon-carbon and hydrocarbon bonds (binding energies 284.8 eV)^40^,^41^,^42^,^43^,^44^ which are also absent on the fresh substrate but can be found after PSE-CVD from Ni(acac)_2_ solution. Photoemission characteristic for C-O bonds can be found as O 1s signal at a binding energy of 530.9 eV (dashed orange “C-O” curve in Fig. 1a) and as C 1s signal with a binding energy of 286.1 eV (dashed orange “C-O” curve in Fig. 1b)^45^,^46^,^47^. Both show the same trend as the water concentration is increased in the 2.5 mM Ni(acac)_2_ precursor solution: slight decrease of the intensity up to 0.5 vol% water and then a significant increase at 4.0 vol% and 10.0 vol% water. Contributions of double C=O bonds can be easily distinguished from C-O emissions only by the C 1s signal (Fig. 1b, dash-dotted green “C=O” curve at 287.9 eV)^46^. In the O 1s emission the C=O signal (dash-dotted green” C=O” curve at 532.9 eV in the Fig. 1a) is somehow overlapped by O 1s emissions from SiOx (Dotted magenta “SiOx” curve with the binding energy 532.1 eV), rendering the separation of the C=O from the SiOx contribution within the O 1s signal difficult.

### PSE-CVD from 2.5 mM solutions

For an interpretation of the XPS results, having an idea of thickness and morphology of the deposits under investigation is instrumental. Supplementary Fig. S1 (online) shows selected SEM data of films deposited from 2.5 mM Ni(acac)_2_ with 0.0 vol%, 0.5 vol%, 5.0 vol%, and 10.0 vol% water in the precursor feedstock, respectively. Within the SEM resolution, all films appear to be continuous or percolated but composed of grains with diameters on the order of some nanometers in the case of 0.0 vol% and 0.5 vol%. Films obtained from precursor solution with 5.0 vol% water show somewhat bigger grains and those deposited from Ni(acac)_2_ solution with 10.0 vol% water show an even coarser morphology with more heterogeneously sized grains in the diameter range of some 10 nanometers.

The thickness of the deposited films was estimated from cross sectional images at a cleaved substrate edge. In contrast to Supplementary Fig. S1, the angle of sample rotation was adjusted in order to obtain a projection along the substrate surface plane (viewing angle 90°) so that the metal layer thickness could be directly measured in the SE micrographs. The measured thickness values are 30 nm, 40 nm, 40 nm, and 50 nm (±5 nm), for films deposited from solutions with 0.0%, 0.5%, 5.0%, and 10.0 vol% water, respectively.

**Figure 2:**
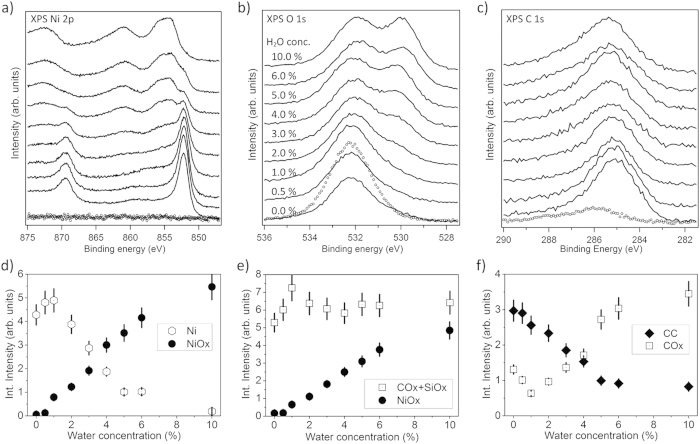
Top panels: XPS spectra in the binding energy range of (a) Ni 2p, (b) O 1s, and (c) C 1s emissions obtained from the SiOx/Si(100) substrate (dotted curves) and from Ni/NiOx films (thickness 30–50 nm) grown ontop by PSE-CVD from 2.5 mM precursor solution with various water concentrations (from 0.0% to 10.0 vol%) at a substrate temperature of 270 °C (solid lines). Bottom panels: Integral peak intensities determined from fits to the XPS data (see text for details).

Figure 2 provides a comprehensive overview of the XPS data in the binding energy regions of a) Ni 2p, b) O 1s, and c) C 1s obtained for films deposited from 2.5 mM Ni(acac)_2_ for various water concentrations in the liquid precursor feedstock mixture. Results of a quantitative analysis of these spectra based on the fitting routine sketched above are compiled in panels d), e), and f). These show the intensities of major signal contributions depending on the water concentration and allow us to pinpoint trends for the composition of the deposited films. Before, it should be noted that, although the film thickness estimates are large with respect to the typical XPS probing depth of few nm, Si 2p emissions, especially those from SiOx, are still present in XP spectra after film deposition. Given the grainy film structure, we assume that density variations and grain boundaries render fingerprints of the substrate–film interface visible in the XPS spectra. In the following, we will discern the two water concentration regimes 0.0% to 1.0 vol% and 1.0% to 10.0 vol% for the discussion and take the waterless solution as reference.

The Ni 2p XP spectrum (Fig. 2d) of the film deposited from waterless precursor solution (0.0 vol% H_2_O) indicates the presence of 97% metallic Ni and only 3% oxidized Ni. When the angle of photoelectron detection is changed to 45° off the surface normal, metallic Ni predominates and accounts for >99.5%, indicating that the oxidized Ni may be located close to the substrate-film interface. Therefore we interpret the Ni-oxide signal as related to Si-O-Ni interlayer species probably forming on the native oxide of the Si wafer (which was not removed prior to deposition as mentioned before) and conclude that, on top of that interlayer, PSE-CVD from waterless precursor solution produces only metallic Nickel. In accordance, we interpret that part of the O 1s emission (Fig. 2e) which can be attributed to Ni-oxide (about 3% of the entire O 1s signal) as related to a Si-O-Ni interlayer. Within error bars, both, the NiOx-related Ni 2p and O 1s signals show the same trends.

It has been reported that M(acac)_x_ precursors are prone to causing relatively high levels of carbon impurities in the deposited films^3^,^7^. For the film deposited from waterless 2.5 mM Ni(acac)_2_ precursor we find the atomic ratio of carbon to nickel (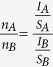, where 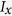 denotes a XPS peak area, and 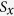 the relative sensitivity factor for element *x*) as about 0.2. This ratio does not change when the polar angle of photoelectron detection is changed to off-normal for higher surface sensitivity. Therefore, we conclude that the carbon is homogeneously distributed within the deposited film. The C 1s photoemission from these carbon impurities is composed of ~70% C-C/C-H and ~30% carbon oxide signals while, in contrast, XPS from intact Cu and Co acetylacetonate precursor clusters had shown a C-C/C-H to COx signal ratio of ~30: 70^48^, i.e., the C-C/C-H fragments of the precursor appear to be less volatile.

Adding a small amount of water (about 0.5% – 1.0 vol%) to the liquid feedstock (all other deposition parameters were left unchanged) leads to significant changes. First, an increase of the absolute intensity of the Ni 2p signal by 31% compared to the waterless solution (Fig. 2d) is observed after PSE-CVD. Given the film thickness well beyond the typical XPS probing depth, we interprete the observed variation of the total Ni 2p intensity as related to surface roughness. As demonstrated by others, an increase of surface roughness leads to a decrease of the total photoemission intensity due to shadowing effects and the reduced off-normal yield from inclined surface regions^49^,^50^,^51^. On the nanometer scale which is not resolved in our SEM data, the admission of up to 1.0 vol% of water to the feedstock probably leads to a smoother surface and a denser film, on the average. Concomitantly, the SEM evaluation indicates that the films grown from precursor feedstock with 0.5 vol% and 1.0 vol% water, respectively, are, on average, thicker than the film grown from waterless solution (40 nm vs. 30 nm), indicating an increase of the growth rate. The NiOx component of the Ni 2p signal which we attributed to a Si-O-Ni interlayer remains almost unchanged when 0.5 vol% water is present in the precursor. It increases significantly only at a water concentration of 1.0 vol% in the precursor and then accounts for more than 13% of the entire Ni 2p signal. Concomitantly, the O 1s emissions associated with NiOx (Fig. 2e) increase at 1.0 vol% of water, accounting then for ~8% of the total O 1s intensity. These step-like increases beyond the photoemissions attributed to a Si-O-Ni interlayer indicate the presence of Ni oxide in the film.

The level of carbon contamination in the deposited films decreases as the water concentration in the liquid precursor feedstock is raised to 0.5% and 1.0 vol%, respectively. For 1.0 vol% water the atomic ratio of carbon to nickel is about 0.1, i.e., half of the value observed for waterless precursor solution, while the C-C/C-H to COx ratio changes to 4:1 (Fig. 2f)^48^.

The O 1s signal that can be related to both, SiOx and COx, linearly increases with the water concentration in the precursor (Fig. 2e) by up to ~40% compared to the waterless solution. The increase observed for 0.5 vol% water is probably due to an increase of the SiOx component, only, as confirmed by the Si 2p signal (not shown here) which reveals that emissions from oxidized Si (Si^2+^) (with a binding energy of 103.3 eV) increase as the water concentration in the liquid feedstock is raised from 0.0 to 1.0 vol%. It should be noted that we do not observe a concomitant increase of the Si^0^ component of the Si 2p signal, indicating that the Si-Ox interlayer grows, probably due to oxidation of the Si substrate by water which, with some caution, may be taken as indicator for the formation of hydroxides at the silica surface upon adsorption of water from the precursor solution^52^,^53^,^54^,^55^. The C 1s signal related to COx (Fig. 2f) even decreases (to ~50% of the intensity found for the waterless precursor) as 1.0 vol% water is added to the precursor solution, supporting the conclusion that the observed increase of the O 1s intensity at a binding energy of 532.9 eV is due to oxidation of the substrate.

In summary, for water concentrations in the range 0.0–1.0 vol% we observed the following trends: Water in the liquid precursor feedstock promotes the growth of metallic Ni and reduces carbon contamination of the deposited films. Ni oxide starts to grow only at water concentrations above 0.5 vol%, so that 0.5 vol% water is optimal for the deposition of fully reduced Nickel films, i.e., water at that concentration has a positive influence on PSE-CVD of Ni films from 2.5 mM Ni(acac)_2_ precursor dissolved in ethanol.

Addition of higher amounts of water (from 1.0% to 10.0 vol%) to the precursor solution causes major changes in the quality and composition of the deposited films. The metallic component in the Ni 2p signal (Fig. 2d) decreases as the water concentration is increased. At the same time the NiOx contribution to the Ni 2p signal and, in accordance, the NiOx contribution to the O 1s signal increase almost linearly with the water concentration. The photoemissions from metallic Ni (Ni^0^) are less than 3% of the total Ni 2p signal at 10.0 vol% of water in the precursor solution, and from the slope of the NiOx signal in Fig. 2d,e, respectively, we assume that almost completely oxidized Ni films are obtained for water concentrations of 8.0 vol% and higher. The films obtained from precursor solution with 5.0 vol% and 10.0 vol% water shown in Supplementary Fig. S1 contain only ~20% metallic Ni and ~80% of the Ni as oxide according to XPS. The total amount of carbon within the deposited films does not change significantly when more than 1.0 vol% water is added to the precursor solution (within error the total intensity of C 1s emissions remains at the same level), but the ratio of C-C/C-H to COx emissions changes from ~4:1 to ~1:4, as the water concentration is increased from 1.0% to 10.0 vol%. Obviously, excessive amounts of water (>1 vol%) in the precursor feedstock lead to oxidation of the deposited Ni and of the carbon impurities in the film.

Overall, while more than 1.0 vol% water in the liquid 2.5 mM Ni(acac)_2_/ethanol feedstock is detrimental for the deposition of Ni films, as it leads to oxidation of the deposited metal, small amounts of water (between 0.0% and 1.0 vol%) have positive effects, such as a lower level of carbon contamination and, apparently, a denser and smoother morphology. An improved growth rate may be linked to a promotion of precursor adsorption on the substrate surface by OH-groups forming at the surface upon adsorption of water, as previously reported for various precursors deposited on alumina, soda-lime glass, and SiO_2_ substrates^4^,^9^,^12^,^14^. On SiO_2_, surface OH-groups have been demonstrated to be stable up to 800 °C and above^12^,^56^,^57^,^58^. At the first nucleation steps of the deposited Ni film, OH-groups on the substrate surface may serve as preferable bonding sites for the precursor^12^,^13^,^14^. Enhancing the initial precursor adsorption rate may be very important in the case of relatively low (2.5 mM) precursor concentrations in the liquid feedstock.

Several studies on the interaction of water vapor with Ni surfaces have been reported^59^,^60^,^61^,^62^,^63^. Oxidation of the Ni surface in presence of water vapor was observed at temperatures as low as 300 °C and even in UHV environment with the oxidation rate increasing as the temperature or the H_2_O dose are increased^59^,^64^. Therefore, as most probable explanation for the onset of Ni oxidation at water concentrations of 1.0 vol% and above we suggest a second decomposition channel of the precursor (where water serves as an oxygen source)^11^, initiated by excessive amounts of water (>3 mol% H_2_O in ~97 mol% C_2_H_5_OH with only 0.01 mol% Ni(acac)_2_ present):


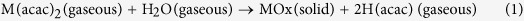


This pathway leads to the formation of Ni oxide and will compete with the precursor decomposition by hydrogen which, as previously shown^30^,^31^, is produced by dehydrogenation of ethanol to acetaldehyde and leads to metallic Ni.

**Figure 3:**
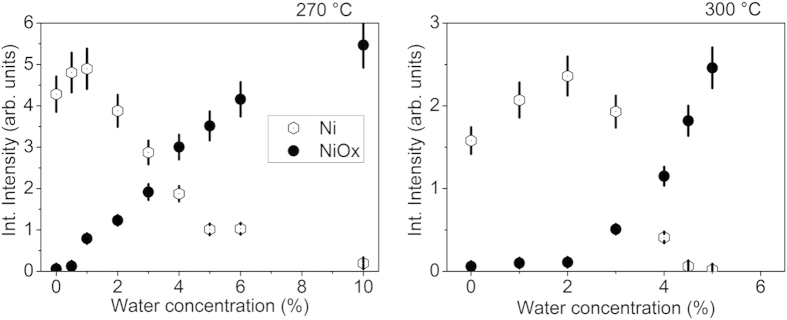
Integral peak intensities determined from fits to the XPS spectra in the binding energy range of Ni 2p emission obtained from the Ni/NiOx films grown ontop SiOx/Si(100) substrate by PSE-CVD from 2.5 mM precursor solution with various water concentrations (from 0.0% to 10.0 vol%) at a substrate temperature of 270 °C (left) and 300 °C (right).

To elucidate how the role of water for the Ni deposition process depends on temperature, PSE-CVD from 2.5 mM Ni(acac)_2_ precursor solution with various water concentrations was also performed at 300 °C substrate temperature which is known to lead to relatively high level of carbon impurities^32^. The analysis of the Ni 2p photoemission spectra is shown in Fig. 3 in direct comparison to the results obtained at 270 °C.

Overall, the integral Ni 2p intensity for the 300 °C deposit is about 50% of that obtained at 270 °C because of carbon contamination which is as high in presence of water as without additional water (data not shown). Similar to the result obtained for 270 °C, a beneficial effect of water on the deposit composition can be identified at 300 °C. There is a maximum of the Ni to NiOx ratio for 2 vol% water which is a four times higher concentration than the optimum for 270 °C. The general trends of the Ni and NiO_x_ contributions below and above the respective maximum, however, are preserved at elevated temperature. Speculating on a role of surface OH groups as precursor adsorption sites, these OH groups are probably removed from the surface at higher rate at elevated temperature. For compensation, at 300 °C a higher water concentration may be needed than at 270 °C in order to reach optimal growth conditions.

### PSE-CVD from concentrated solutions

To elucidate the interplay between all three components of the precursor solution, i.e. Ni(acac)_2_, ethanol and water, PSE-CVD at varying water concentration in the feedstock was also performed starting with 5.0 mM and 7.5 mM concentrations of Ni(acac)_2_ in ethanol, respectively. As before, the total deposition time was kept constant at 30 min for all deposits.

**Figure 4:**
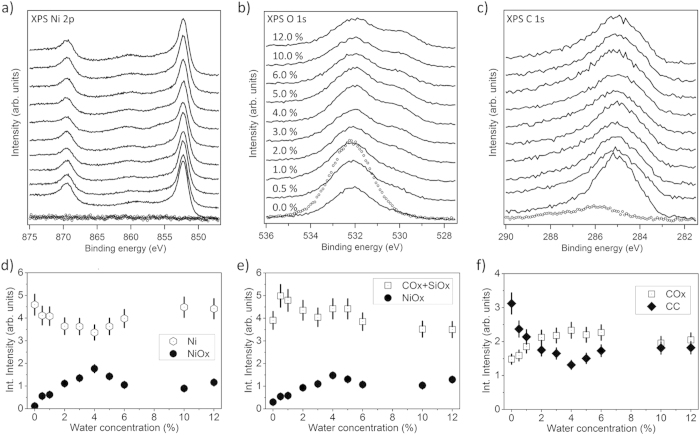
Top panels: XPS spectra in the binding energy range of (a) Ni 2p, (b) O 1s, and (c) C 1s emissions obtained from the SiOx/Si(100) substrate (dotted curves) and from Ni/NiOx films (thickness 60–120 nm) grown ontop by PSE-CVD from 5.0 mM precursor solution with various water concentrations (from 0.0% to 12.0 vol%) at a substrate temperature of 270 °C (solid lines). Bottom panels: Integral peak intensities determined from fits to the XPS data (see text for details).

Qualitatively, the data obtained for 5.0 mM and 7.5 mM solutions show very similar trends when compared to each other while they show significant differences to the data obtained for the 2.5 mM solution. Figure 4 depicts the dependence of XPS in the binding energy regions of a) Ni 2p, b) O 1s, and c) C 1s on water concentration in a liquid feedstock solution of 5.0 mM Ni(acac)_2_ in ethanol. Similar to Fig. 2, the quantitative analysis of these spectra is condensed into plots d), e), and f), so that the dependence of the deposited Ni film composition on water concentration in the precursor can be followed in detail. For the discussion, the two water concentration ranges 0.0 to 4.0 vol% water and 4.0 to 12.0 vol% water are discerned and, again, the waterless solution is taken as reference.

The Ni 2p photoemission spectrum of a film deposited from waterless 5.0 mM Ni(acac)_2_ solution (Fig. 4d) shows ~99% metallic Ni and only about 1% oxidized Ni which we attribute to a Si-O-Ni interlayer, in accordance with the results obtained for 2.5 mM precursor solution. Here we take into account that a thicker film has grown from 5.0 mM Ni(acac)_2_ (60 nm as will be demonstrated below) which should lead to stronger attenuation of the interlayer XPS signal.

In contrast to PSE-CVD with 2.5 mM precursor solution, pure metallic Ni films can be obtained from 5.0 mM Ni(acac)_2_ precursor dissolved in ethanol only without additional water (0.0 vol%), a positive influence of small amounts of water on the growth rate was not observed. With respect to carbon contamination levels we find C 1s intensities after Ni film deposition from waterless 5.0 mM Ni(acac)_2_ precursor similar to the deposition from waterless 2.5 mM precursor. The total C 1s to Ni 2p atomic ratio is about 0.2, while carbon impurities (Fig. 4f) consist of ~70% C-C/C-H and 30% C-O/C=O.

When up to 4.0 vol% water are added to the 5.0 mM Ni(acac)_2_ solution, the film composition significantly changes. The Ni 2p signal representing the metallic fraction of deposited Ni (Fig. 4d) decreases linearly as the H_2_O concentration is increased (that also holds for 0.5 vol% water). Instead, a NiOx component becomes evident in O 1s and Ni 2p, accounting for 34% of the entire Ni 2p signal at maximum. Also for PSE-CVD from 5.0 mM Ni(acac)_2_ precursor solution the addition of water promotes reduction of the carbon impurity level in the film and we found an atomic ratio C : Ni of ~0.14 at minimum by comparing the C 1s an Ni 2p emissions. In contrast to PSE-CVD from 2.5 mM precursor solution, however, the oxidation of carbon impurities sets in already at very low water concentrations as the COx signal in Fig. 4f indicates. The strong singular increase of the combined SiOx and COx components of the O 1s emission upon adding 0.5 vol% water to the waterless precursor solution indicates that, similar to deposits obtained from 2.5 mM precursor, water promotes oxidiation of the Si substrate (Si-Ox interlayer growth).

When the water concentration in the liquid feedstock is increased beyond 4.0 vol%, the Ni oxide content in deposited films does not increase further but even slightly decreases as the water content is raised, so that for water concentrations of ~10 vol% and higher the metallic component of the Ni 2p signal saturates at a level of about 80% of the total nickel emission. As Fig. 4f shows, water in excess of 4.0 vol% in 5.0 mM Ni(acac)_2_ solution does not change the total level of carbon contamination in the deposited film and, in contrast to the 2.5 mM precursor solution, has no significant influence on carbon oxidation. At water concentrations of 10.0 vol% and higher the ratio between COx and the C-C/C-H contributions to the entire C 1s signal is about 1:1.

**Figure 5:**
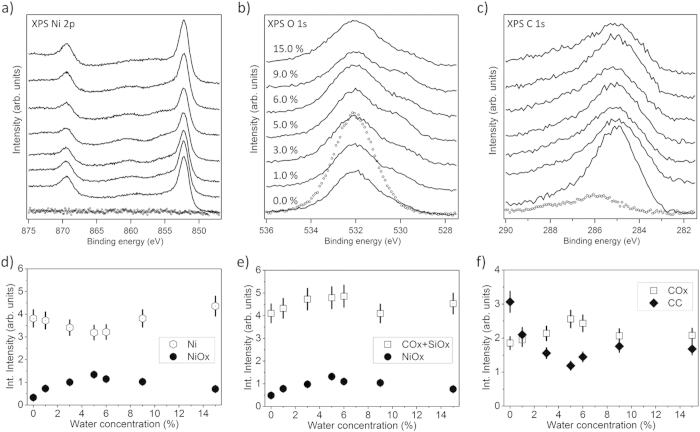
Top panels: XPS spectra in the binding energy range of (a) Ni 2p, (b) O 1s, and (c) C 1s emissions obtained from the SiOx/Si(100) substrate (dotted curves) and from Ni/NiOx films (thickness 120–220 nm) grown on top by PSE-CVD from 7.5 mM precursor solution with various water concentrations (from 0.0% to 15.0 vol%) at a substrate temperature of 270 °C (solid lines). Bottom panels: Integral peak intensities determined from fits to the XPS data (see text for details).

The XPS data obtained for PSE-CVD of Ni from 7.5 mM Ni(acac)_2_ precursor solution are compiled in Fig. 5 and show the same trends within the water concentration ranges 0.0–5.0 vol% and 5.0–15.0 vol%, respectively, as discussed for the deposits from 5.0 mM precursor solution with 0.0–4.0 vol% and 4.0–12.0 vol% water, respectively. Almost metallic Ni films (92% Ni^0^, Fig. 5d) can be obtained from 7.5 mM precursor solutions only without additional water (0.0 vol%). The Ni oxide content in the film is maximal for 5.0 vol% of water in the feedstock and then accounts for 30% of the total Ni 2p XPS signal. For the waterless 7.5 mM precursor solution carbon impurities in the deposited film (Fig. 5f) consist of ~60% C-C/C-H species and ~40% carbon oxides according to the C 1s signal, with the total amount of contamination (atomic ratio C 1s to Ni 2p is ~0.23) being somewhat higher than in films deposited from 2.5 and 5.0 mM solutions. In contrast to the findings for 2.5 and 5.0 mM precursor solutions, the O 1s signal attributed to SiOx and COx components (Fig. 5e) does not increase as 0.5 vol% water is added to the waterless 7.5 mM precursor solution. Concomitantly, however, the Si 2p substrate signal (not shown here) is not detected on the deposited films due to the large film thickness (>120 nm, see below) so that XPS is not expected to provide any evidence for water-induced oxidation of the Si-Ox interlayer.

SEM images of films deposited from 5.0 mM Ni(acac)_2_ precursor solution with 0.0 vol% and 12.0 vol% water, respectively, are shown in Supplementary Fig. S2 (online). The film surfaces obtained from the waterless feedstock appear to be continuous and relatively smooth, in contrast to films obtained from solution with 12.0 vol% water (~80% Ni and ~20% NiO), which show a coarse morphology with separate, relatively large and heterogeneously sized grains (grain diameters from some 10 nm up to 150 nm). Compared to the films obtained from 2.5 mM precursor solution, the deposits from 5.0 mM solution have bigger grains and very significant water-induced changes of morphology. The average film thickness measured by SEM is about 60 nm, 70 nm, and 120 nm (±5 nm) for films deposited from solutions with 0.0%, 5.0%, and 12.0 vol% water, respectively.

Nickel films deposited from waterless 7.5 mM Ni(acac)_2_ show an even coarser morphology (Supplementary Fig. S3) than the films obtained from waterless 5.0 mM solution, but with homogeneously sized grains. While films obtained from solution with 5.0 vol% water shows somewhat bigger grains, SEM of the film deposited from 7.5 mM solution with 15.0 vol% water reveals a very coarse morphology, with grain diameters from ~50 nm up to 150 nm.

The measured average thickness values are 120 nm, 200 nm, and 220 nm, approximately, for films deposited from solutions with 0.0%, 5.0%, and 15.0 vol% water, respectively. Apparently, the precursor decomposition rate is unaffected by the amount of water within the water concentration range 5.0% to 15.0 vol% as about the same film thickness is obtained. The density of Ni/NiOx nuclei, however, decreases significantly as the water concentration is increased from 5.0% to 15.0 vol% of water, leading to fewer but much bigger grains. Obviously, excessive amounts of water in the precursor limit the nucleation rate of the growing film. At this point we can only speculate that the reportedly low reactivity of β-diketonate precursors towards surface groups^14^ may hinder the initial Si-O-Ni bond formation between precursor and an OH group–saturated substrate, thereby delaying the formation of nuclei which, at high precursor concentrations in the liquid feedstock and on the surface, then quickly grow.

In summary, we observe no positive effect of water on the growth rate or carbon impurity levels of metallic Ni films for 5.0 and 7.5 mM Ni(acac)_2_ feedstock, in contrast to deposition from 2.5 mM precursor solutions. We presume that any promotional effect of surface OH-groups for the precursor adsorption should be most efficient at lowest precursor concentrations whereas at 5.0 mM and 7.5 mM precursor concentration in the deposited gas phase (spray) the precursor molecule density on the surface may be so high that there is no need to promote adsorption.

An intriguing observation is the fact that at high precursor concentrations (5.0 mM and 7.5 mM) the Ni oxide fraction within the film is maximum for an intermediate water concentration of 4.0% – 5.0 vol% and decreases as the H_2_O concentration is further increased. We presume that the morphology of the growing films provides a key to this puzzle: While it seems clear that water promotes the oxidation of Ni, excessive amounts of water do also limit the nucleation rate and promote the growth of existing nuclei (see above), leading to only few and but larger grains within the film. Due to the lower surface-to-volume ratio, however, large metal grains are presumably less efficiently oxidized than metal films with small grains^65^,^66^, limiting the total oxide fraction in the deposited film. Our observation that films deposited from 2.5 mM solution with small grains (<5 nm) can be completely oxidized in the presence of water while films with larger grains (as deposited from 5.0 and 7.5 mM solutions and high H_2_O concentration) can be oxidized only up to a maximum fraction of ~30% is in accordance with this presumption.

Overall, our results show that by tuning the concentrations of Ni(acac)_2_ and water in the precursor solution, the fraction of Ni metal and Ni oxide in the film or the film morphology can be adjusted. At low (2.5 mM) precursor concentration in the feedstock smoothest morphologies (with grains from 5 to 10 nm) are obtained and the fraction of Ni oxide in the deposit is adjustable from 0 to 100% by varying the water concentration. At high Ni(acac)_2_ concentrations (5.0 and 7.5 mM) excessive amounts of water strongly affect the film morphology and grain sizes can be varied from 10 to 150 nm. The carbon contamination in deposited films can be minimized to less than 10% when the precursor solution is adjusted to 2.5 mM Ni(acac)_2_ concentration and 0.5–1.0 vol% water content.

### Co(acac)_2_ in EtOH on SiOx/Si(100)

**Figure 6:**
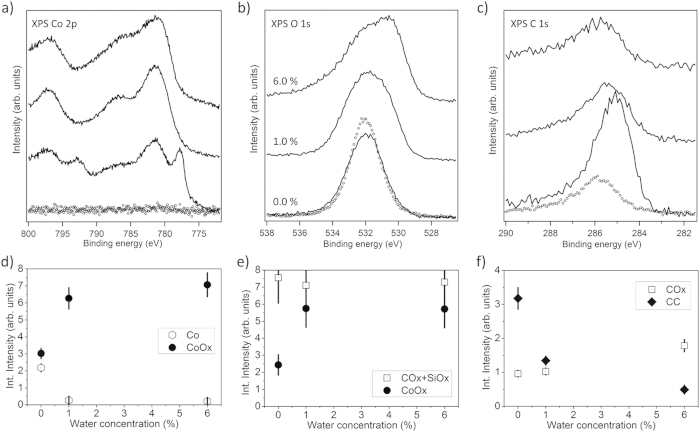
Top panels: XPS spectra in the binding energy range of (a) Co 2p, (b) O 1s, and (c) C 1s emissions obtained from the SiOx/Si(100) substrate (dotted curves) and from Co/CoOx films (thickness 100–150 nm) grown ontop by PSE-CVD from 7.5 mM precursor solution with various water concentrations (from 0.0% to 6.0 vol%) at a substrate temperature of 310 °C (solid lines). Bottom panels: Integral peak intensities determined from fits to the XPS data (see text for details).

XPS data from films obtained by PSE-CVD from 7.5 mM Co(acac)_2_ in ethanol on SiOx/Si(100) were collected and analyzed in a similar way as shown in the previous section for Ni deposits and compiled into Fig. 6. Co 2p spectra were fitted by contributions from Co^0^ and Co^2+^ emissions similar to previous reports^67^,^68^. In contrast to nickel, the deposits from Co(acac)_2_ contained mainly CoOx, even when waterless precursor solutions were used. With increasing precursor concentration from 2.5 mM (data not shown) to 7.5 mM the Co metal fraction significantly increased, but accounted for only 42% of the Co 2p signal (Fig. 6d).

For the film deposited from waterless 7.5 mM Co(acac)_2_ precursor we find the atomic ratio of Carbon to Cobalt as 0.18. The C 1s signal (Fig. 6f) is composed of 77% C-C/C-H and 23% COx species. That component of the O 1s signal which can be attributed to CoOx is about 34% of the entire O 1s signal. Because the O 1s signal from SiOx is quite strong, we may assume that Co films are porous. (Supplementary Fig. S4 shows all contributions that were considered for fitting the O 1s spectra.) Adding a small amount of water (about 1.0 vol%) to the precursor solution leads to significant decreases of the metallic component in the Co 2p signal (Fig. 6d): Co^0^ accounts only for 4% of the entire Co 2p signal (96% Co^2+^) at 1.0 vol% of water in the precursor solution. The Co^0^: Co^2+^ ratio does not change much at even higher water concentrations (Co 2p accounts for 3% metallic cobalt and 97% cobalt oxide at 6.0 vol% water), i.e., essentially only CoOx is grown. Compared to the films grown from waterless feedstock, the total level of carbon contamination in the film (total C 1s intensity) decreases to ~50–60% as water is added to the precursor solution while the ratio of the COx and C-C/C-H component indicates oxidation of the carbon impurities by water as it increases from ~1:1 at 1.0 vol% water to ~4 : 1 at 6.0 vol% water in the precursor solution (Fig. 6f).

SEM reveals that the surface of the Co films deposited from waterless 7.5 mM Co(acac)_2_ (42% Co^0^, 60% Co^2+^ according to XPS) exhibit a coarse morphology and heterogeneously sized grains. The structure visible in Supplementary Fig. S5 available online (bright grains with diameters of ~10 nm within a greyish matrix) may indicate separation of (metallic) Co particles and Co oxide. In fact, the Co oxide films obtained from solution with 6.0 vol% water do not show clear grains but appear somehow fluffy. The film thickness determined by SEM is about 100 nm and 150 nm for films deposited from 7.5 mM Co(acac)_2_ solutions with 0.0% and 6.0 vol% water, respectively.

In summary, in contrast to PSE-CVD from Ni(acac)_2_ even small amounts (1.0 vol%) of water in the 7.5 mM CO(acac)_2_ precursor feedstock lead to complete oxidation of the deposited Co. A significant fraction of metallic Co^0^ can be detected in the Co 2p signal only in deposits obtained without water. As well known from recent reports, cobalt is not as good a catalyst for ethanol dehydrogenation as Nickel is^31^,^34^. Therefore, even small amounts of water in the feedstock probably lead to a high water/hydrogen ratio at the substrate surface, rendering the precursor decomposition and CoOx formation according to Eq. 1 as dominant pathway^11^ because Co reduction by hydrogen is expected to be much slower. In accordance with observations made for Ni deposition, we find also water-induced oxidation of carbon impurities when the reaction mechanism changes from M to MOx formation. A significant metallic fraction of deposited Co is observed only for the waterless precursor solution, the observed maximum ratio of Co : CoOx is ~40 : 60.

**Figure 7:**
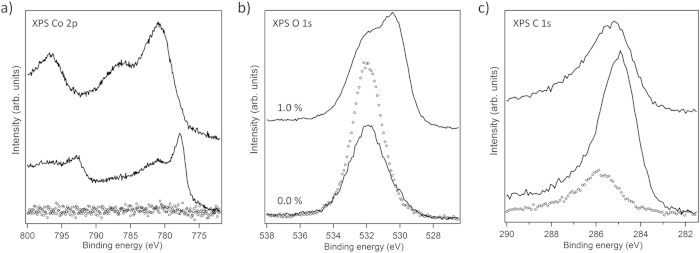
XPS spectra in the binding energy range of (a) Co 2p, (b) O 1s, and (c) C 1s emissions obtained from the Ni/SiOx/Si(100) substrate (dotted curves) and from Co/CoOx films (thickness 100–150 nm) grown ontop by PSE-CVD from 7.5 mM precursor solution with 0.0% and 1.0 vol% water concentrations at a substrate temperature of 310 °C (solid lines).

Because previous studies demonstrated that a Ni interlayer on the SiOx/Si substrate may drastically improve the quality of films deposited from Co(acac)_2_ on top (almost completely metallic Co, only small amount of CoOx)^31^,^34^, we also included PSE-CVD from 7.5 mM Co(acac)_2_ on a 40 nm thick Ni layer on SiOx/Si(100) in our experiments. The results obtained for a waterless precursor solution and for 1.0 vol% water in the feedstock are shown in Fig. 7. For deposits from the waterless solution, the Co^2+^ emission (Fig. 7a) accounts for 33% of the entire Co 2p signal which is significantly lower than the oxide fraction detected from films deposited without the Ni interlayer (Fig. 6a). But also with a Ni interlayer, even small amounts (1.0 vol%) of water in the feedstock lead to complete oxidation of the deposited Co, as the Co 2p signal clearly shows. Consequently, in order to benefit from a Ni seed layer for the PSE-CVD of metallic Co from Co(acac)_2_ in ethanol, care has to be taken that the precursor solution is kept waterless. As mentioned before, previous publications^31^,^34^ suggest that the benefit of a Ni seed layer is catalysis of ethanol dehydrogenation for the formation of hydrogen as reducing agent. Co and in particular CoOx are worse catalysts in this respect. Consequently, while a Ni layer helps to catalyse the formation of an initial metallic Co layer which then may sustain the Co^0^ formation on top by catalyzing ethanol dehydrogenation, the balance quickly shifts to the precursor decomposition pathway depicted in Eq. 1 as soon as water is present because water oxidizes deposited Co which, in turn, is not available to catalyze ethanol dehydrogenation.

## Summary

Employing PSE-CVD with liquid feedstock of Ni(acac)_2_ or Co(acac)_2_ dissolved in ethanol, systematic studies of the influence of water on growth and composition of Ni and Co metal and oxide films were performed. The ethanol-based liquid precursor enabled us to control and vary the water concentration during the deposition process easily and reliably within a range from 0.0 up to 15.0 vol% with an uncertainty of only 0.1%. Using a specially designed CVD reactor directly attached to a UHV system, *in vacuo* transfer of samples after any stage of deposition rendered possible a quasi *in situ* analysis of to film composition by XPS.

Both, beneficial and detrimental effects of water on the growth of metallic films, strongly depending on the concentration of water and the β-diketonate in the precursor solution, were observed. The smoothest morphologies (with grains from 5 to 10 nm) were obtained for metallic Ni films grown from 2.5 mM Ni(acac)_2_ precursor solution. The film growth could be optimized and the carbon contamination of the deposited Ni films minimized to less than 10% when the water concentration in the precursor solution was adjusted to 0.5 vol%. Only at concentrations of 1.0 vol% and higher water induced significant oxidation of the Ni deposit. From precursor solutions with higher Ni(acac)_2_ concentrations (5.0. and 7.5 mM), purely metallic films were only obtained when any water in the feedstock was eliminated. Here, the film morphology strongly depended on the water concentration, indicating inhibition of nucleation on the Si/SiOx substrate by excessive amounts of water which lead to the growth of relatively large grains and a coarse film morphology. Overall, the gathered detailed overview over water-induced effects on the PSE-CVD with Ni acetylacetonate precursors dissolved in ethanol shows that by carefully tuning the precursor and water concentration in the feedstock, e.g., the degree of Ni oxidation can be adjusted from 0 to 100% or the size of the grains the film is composed of can be varied from less than 10 to 150 nm.

Compared to Ni(acac)_2_, an even higher sensitivity for water in the feedstock was observed in the case of Co(acac)_2_ as precursor. Significant metallic Co fractions within the deposited films on SiOx/Si(100) as well as on Ni/SiOx/Si(100) were obtained only from waterless solutions. The relative sensitivities of Ni- and Co-based precursor solutions on water indicate a strong competition between two precursor decomposition pathways, one probably involving the Ni- or Co-catalyzed dehydrogenation of ethanol and H_2_-induced reduction of the precursor metal center, the other involving H_2_O-induced Ni or Co(acac)_2_ decomposition which leads to Ni or Co oxidation.

## Additional Information

**How to cite this article**: Weiss, T. *et al.* Influence of Water on Chemical Vapor Deposition of Ni and Co thin films from ethanol solutions of acetylacetonate precursors. *Sci. Rep.* **5**, 18194; doi: 10.1038/srep18194 (2015).

## Supplementary Material

Supplementary Information
