# Supplementary material for: Influence of Water on Chemical Vapor Deposition of Ni and Co thin films from ethanol solutions of acetylacetonate precursors
Source: Sci Rep. 2015 Dec 14;5:18194. doi: 10.1038/srep18194 (PMC4677359; doi:10.1038/srep18194)
Supplement: Supplementary Information [file srep18194-s1.pdf]

Supplementary Information

Influence of Water on Chemical Vapor Deposition of Ni and Co thin films from ethanol solutions of acetylacetonate precursors

Theodor Weiss, Volkmar Zielasek*, Marcus Bäumer

Institut für Angewandte und Physikalische Chemie, Universität Bremen, Leobener Straße UFT, D-28359 Bremen, Germany

*) corresponding author

Experimental Parameters

Supplementary Table S1. PSE-CVD reactor parameters for Ni and Co metal films deposition

Precursor:	$Ni(acac)_2$	$Co(acac)_2$
Solvent:	Ethanol	
Concentration of the precursor [mmol/l]	2.5, 5.0, 7.5	7.5
Water concentration [vol%]	0.0 – 15.0	
Spray pulse frequency [Hz]	2	
Spray pulse width [ms]	15	
Deposition area pressure [Pa]	$5 \cdot 10^3$	
Carrier gas flow rate [sml/min]	500	
Substrate temperature [° C]	270	310
Total duration of deposition [min]	30	

Scanning Electron Micrographs of Ni deposits

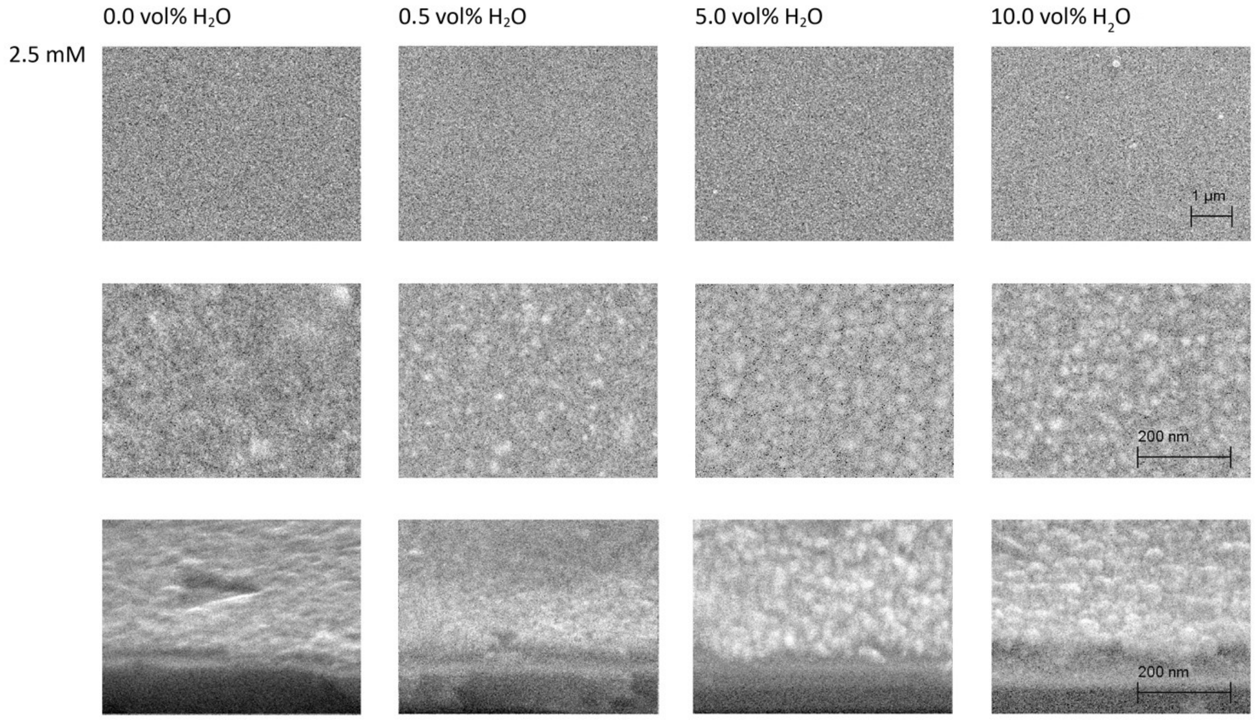

Supplementary Figure S1. SEM of films deposited on a SiOx/Si(100) substrate from 2.5 mM Ni(acac)$_2$ in ethanol with 0.0 %, 0.5 %, 5.0 %, and 10.0 vol% water, respectively. First row: plane view on the substrate surface (scale bar 1 μm); second row: enlarged plane view on substrate surface (scale bar 200 nm); third row: enlarged view (scale bar 200 nm) at a viewing angle of ~ 60° with respect to surface normal, showing a cleaved sample edge with the substrate (bottom) and deposits on top. Film thicknesses estimated by SEM: 30 nm, 40 nm, 40 nm, and 50 nm for films obtained from solutions with 0.0 %, 0.5 %, 5.0 % and 10.0 vol% of water, respectively.

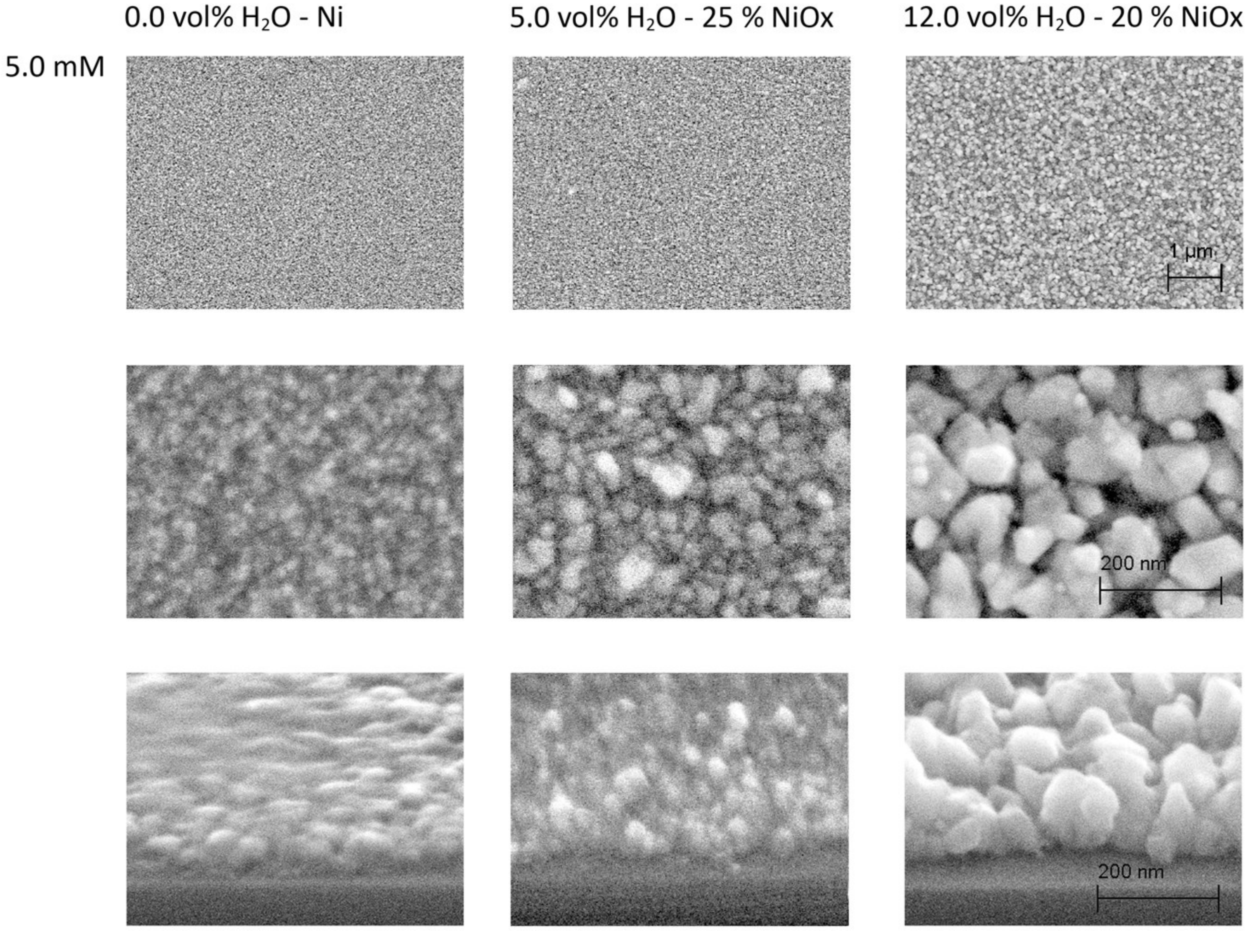

Supplementary Figure S2. SEM of Ni/NiOx films deposited on a SiOx/Si(100) substrate from 5.0 mM $Ni(acac)_2$ in ethanol with 0.0 % and 12.0 vol% water, respectively. First row: plane view on the Ni-covered substrate surface (scale bar 1 μm); second row: enlarged plane view on the Ni-covered substrate surface (scale bar 200 nm); third row: enlarged view (scale bar 200 nm) at a viewing angle of ~ 60° with respect to surface normal, showing a cleaved sample edge with the substrate (bottom) and Ni deposits on top. Film thicknesses estimated by SEM: 60 nm, 70 nm, and 120 nm for Ni/NiOx films obtained from solutions with 0.0 %, 5.0 %, and 12.0 vol% of water, respectively.

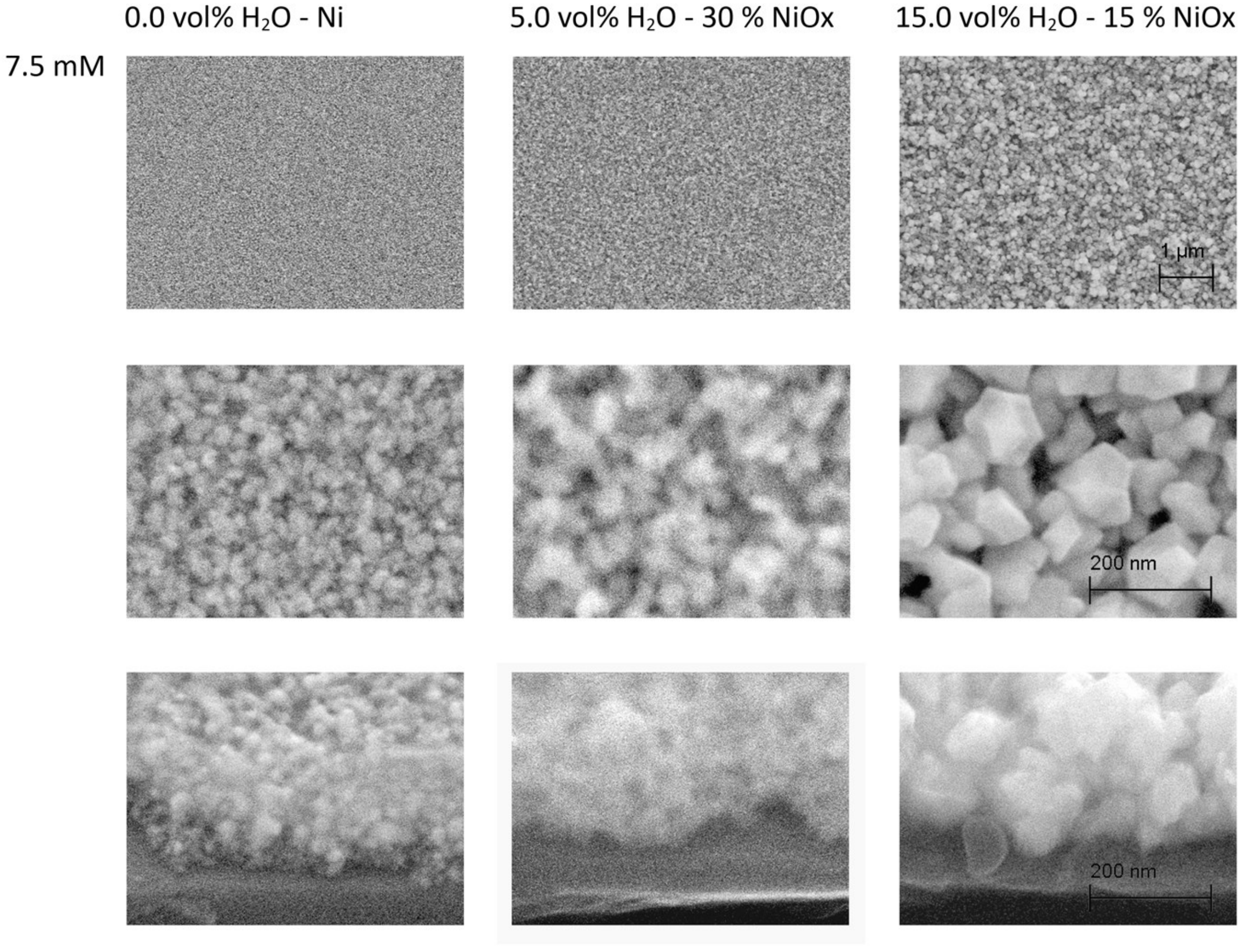

Supplementary Figure S3. SEM of Ni/NiOx films deposited on a SiOx/Si(100) substrate from 7.5 mM Ni(acac)$_2$ in ethanol with 0.0 %, 5.0 %, and 12.0 vol% water, respectively. First row: plane view on the Ni-covered substrate surface (scale bar 1 μm); second row: enlarged plane view on the Ni-covered substrate surface (scale bar 200 nm); third row: enlarged view (scale bar 200 nm) at a viewing angle of ~ 60° with respect to surface normal, showing a cleaved sample edge with the substrate (bottom) and Ni deposits on top. Film thicknesses estimated by SEM: 120 nm, 200 nm, and 220 nm for Ni/NiOx films obtained from solutions with 0.0 %, 5.0 %, and 12.0 vol% of water, respectively.

X-ray Photoelectron Spectra of Co deposits

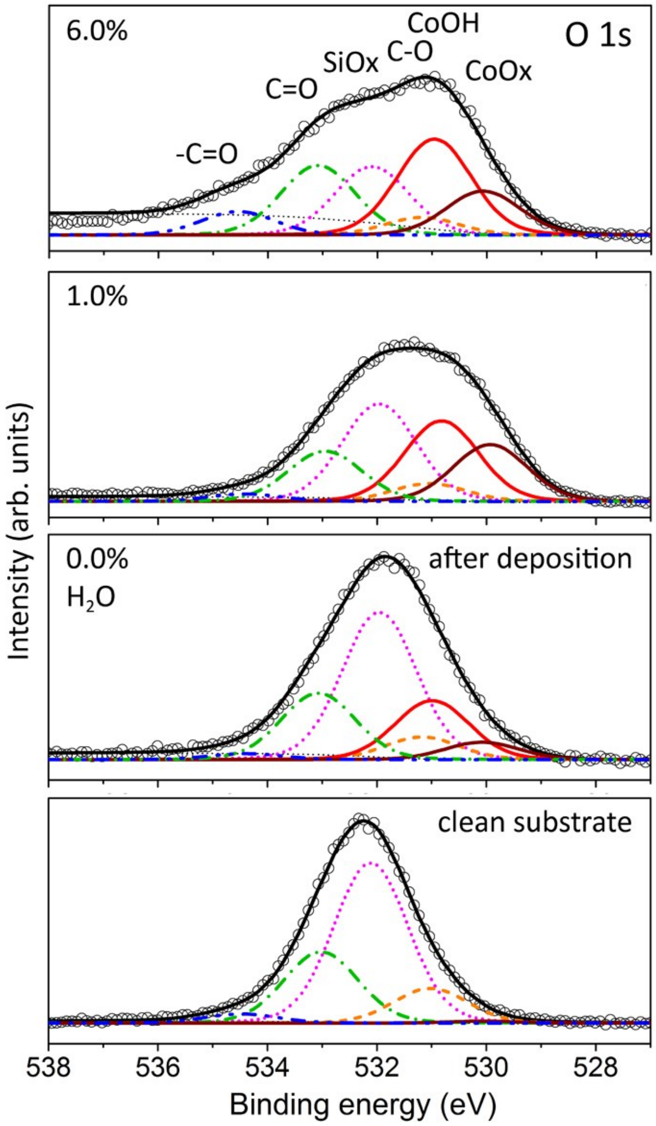

Supplementary Figure S4. Fits of the O 1s XPS spectra obtained from the SiOx/Si(100) substrate (bottom panel) and Co/CoOx films grown ontop by PSE-CVD from 7.5 mM Co(acac)$_2$ in ethanol and various water concentrations (0.0 %, 1.0 %, and 6.0 vol%) in the precursor solution at a substrate temperature of 310 °C.

Scanning Electron Micrographs of Co deposits

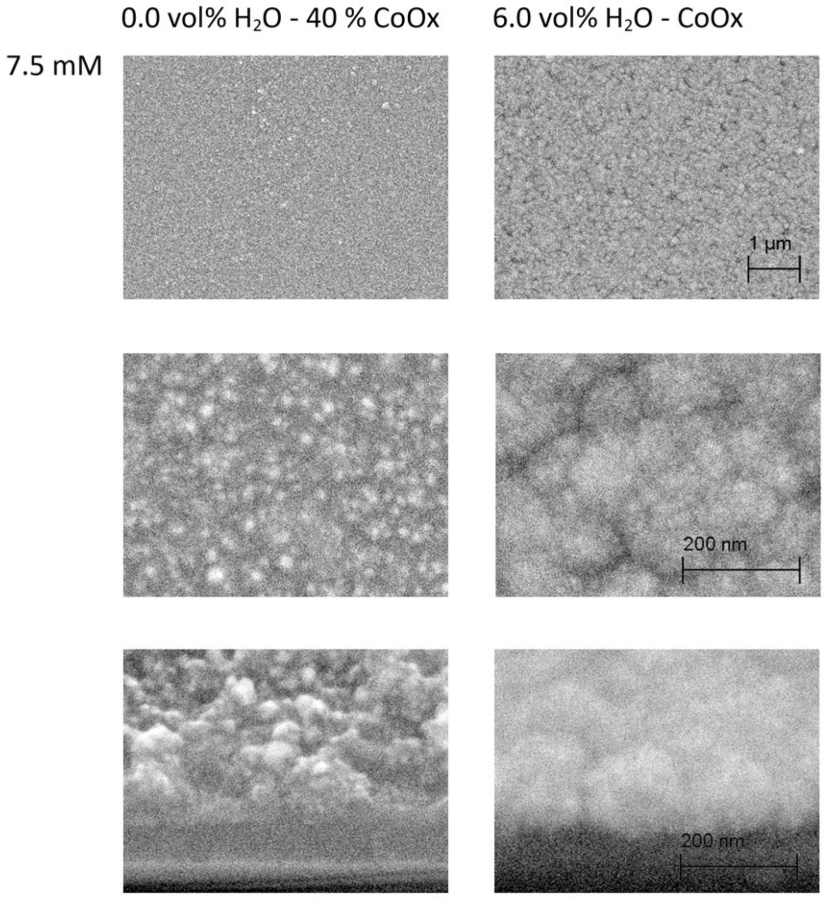

Supplementary Figure S5. SEM of Co/CoOx films deposited on a SiOx/Si(100) substrate from 7.5 mM $Co(acac)_2$ in ethanol with 0.0 % and 6.0 vol% water, respectively. First row: plane view on the Co-covered substrate surface (scale bar 1 µm); second row: enlarged plane view on the Co-covered substrate surface (scale bar 200 nm); third row: enlarged view (scale bar 200 nm) at a viewing angle of ~ 60° with respect to surface normal, showing a cleaved sample edge with the substrate (bottom) and Co deposits on top. Film thicknesses estimated by SEM: 100 nm and 150 nm for Ni/NiOx films obtained from solutions with 0.0 % and 6.0 vol% of water, respectively.
